# Millisecond flash lamp curing for porosity generation in thin films

**DOI:** 10.1038/s41598-023-34748-x

**Published:** 2023-05-12

**Authors:** Ahmed G. Attallah, Slawomir Prucnal, Maik Buttering, Eric Hirschmann, Nicole Koehler, Stefan E. Schulz, Andreas Wagner, Maciej O. Liedke

**Affiliations:** 1 Institute of Radiation Physics, Helmholtz-Zentrum Dresden-Rossendorf, 01328 Dresden, Germany; 2 Physics Department, Faculty of Science, Minia University, Minia, 61519 Egypt; 3 Institute of Ion Beam Physics and Materials Research, Helmholtz-Zentrum Dresden-Rossendorf, 01328 Dresden, Germany; 4 Center for Microtechnologies, Chemnitz University of Technology, 09107 Chemnitz, Germany

**Keywords:** Materials science, Materials for devices, Nanoscale materials

## Abstract

Flash lamp annealing (FLA) with millisecond pulse durations is reported as a novel curing method for pore precursor's degradation in thin films. A case study on the curing of dielectric thin films is presented. FLA-cured films are being investigated by means of positron annihilation spectroscopy (PAS) and Fourier-transform infrared (FTIR) spectroscopy in order to quantify the nm-scale porosity and post-treatment chemistry, respectively. Results from positron annihilation reveal the onset of the formation of porous voids inside the samples at 6 ms flash treatment time. Moreover, parameter's adjustment (flash duration and energy density) allows for identifying the optimum conditions of effective curing. Within such a systematic investigation, positron results indicate that FLA is able to decompose the porogen (pore precursors) and to generate interconnected (open porosity) or isolated pore networks with self-sealed pores in a controllable way. Furthermore, FTIR results demonstrate the structural evolution after FLA, that help for setting the optimal annealing conditions whereby only a residual amount of porogen remains and at the same time a well-densified matrix, and a hydrophobic porous structures are created. Raman spectroscopy suggests that the curing-induced self-sealing layer developed at the film surface is a graphene oxide-like layer, which could serve as the outside sealing of the pore network from intrusions.

## Introduction

Since decades, the development of porous materials has been a fascinating research topic^1^. Porous materials are defined as solids that possess voids or pores, which lie in three categories; micropores (pore size < 2 nm), mesopores (pore size 2–50 nm), and macropores (pore size > 50 nm)^2^. The ability to create smaller and smaller holes and control their arrangement has resulted in the development of a variety of new materials that are now utilized in everyday or industrial applications such as catalysis^3,4^, separation science^5^, energy storage^6^, microelectronics^7,8^, and biomedical engineering^9,10^. There is always a trade-off between porosity and the final material's physical properties in every application^11^. Therefore, it is necessary to optimize the range of porosity, pore morphology, and pore size distribution for particular applications. Depending on the fabrication technique chosen, this is controlled and determined. In the design of materials, the fabrication process necessitates strict guidelines and meticulous planning in order for the finished product to satisfy a variety of applications, such as stability, custom structure, reusability, cost-effectiveness, etc. A class of porous materials that requires a proper porous design is the low-k dielectric thin films used as interlayer insulators in microelectronic applications^12,13^. For instance, during the fabrication of low-k thin films, large and interconnected pores result in observable plasma-induced damages that cause charges to build up and ultimately increase the leakage current^14^. Furthermore, porous low-k dielectrics with interconnected pores have poor mechanical properties, which complicates their incorporation into microchips^15^. Another challenge in low-k dielectric thin films is the open-to-surface pore network, which will allow moisture and other contaminants to enter the porous network, degrading the k-value. Therefore, controlling over the porosity architecture is important from the performance and integration perspectives. This includes a closer look at how the pores are being formed and how to tune this formation process in order to get desirable porous films.

Porogens (pore precursors) are often used as additives to form pores in low-k thin films^16,17^. Porogens are tiny molecules or polymers that are added to the precursor solution prior to the formation of the thin film. These molecules are engineered to be easily removed after the thin film has been produced, leaving gaps in the thin film^18^. A solvent, for example, can be used as a porogen then evaporated, leaving pores behind. External stimuli (heat, light, etc.) are used to eventually cause the porogen to evaporate^19^. These porogen removal methods are notorious for producing interconnected pores, which may extend towards the film surface^20^. For example, the relatively low ramping rates of ~ 10 K/min^20,21^ during thermal curing at 400–450 °C allows for agglomerating the porogen, whereby interconnected and open-to-surface pores are developed^20^. Similarly, plasma- and UV–thermal assisted methods, despite their short curing time (~ few minutes)^22^ and reduced thermal budget^23^, leave interconnected pores^24^, which often limits the versatility of these approaches. Agglomeration occurs when small pores in the material merge together to form larger pores, resulting in a reduction in the total number of pores and an increase in the average pore size. This can lead to a decrease in the specific surface area and an increase in the tortuosity of the material. Additionally as discussed above, it can also negatively influence the mechanical properties of the material, such as its strength and durability leading to a reduction in its lifespan.

Faster curing rates, on the other hand, where the curing process is accelerated to occur within a much shorter period of time, tend to vitrify the matrix rapidly and prevent or at least decrease the porogen diffusion and clustering^20,21,25^. In such a way, closed and isolated pores could be formed and porous materials with improved chemical and physical properties would be manufactured. Millisecond Laser spike annealing (LSA) was introduced to low-k processing as a fast post-curing treatment^26,27^. LSA has been utilized as a post-curing process in order to enhance the mechanical stability by oxidative or bond redistribution transformations^27^ or by retaining the stability of ethyl bridge structures within the silica network^26^. Prior to LSA in these studies, spin-on coated dielectric films have been cured at 400 °C for 1 h^26^ or at 450 °C for 2 h^27^. Despite the improved mechanical stabilities of the LSA-treated low-k films, the initial thermal curing at 400–450 °C is believed to form interconnected pores^20^ with the drawbacks mentioned above.

The preceding discussion has convinced us that an extremely faster curing process may be used as an appropriate curing method in fields requiring isolated pores by preventing the pore-forming agents from clustering. In this regard, the current work proposes an alternative millisecond curing approach; the so-called flash lamp annealing (FLA)^28^ as a one-shot curing method. In FLA, the film surface is treated with one or more energetic flashes of a flash lamp. Thus, material characteristics can be altered without thermally stressing the substrate below. So far, FLA is employed for short-time annealing applications ranging from semiconductor doping to the treatment of polymers and flexible substrates^28^. Worth mentioning, the focus of this work is to demonstrate the capability of FLA to decompose the porogen and create porosity. Here, we use curable dielectric thin films as a case study to validate the method without discussing the dielectric or mechanical properties of the resulting porous films. For the first time, to the best of our knowledge, FLA is used as a stand-alone approach to degrade porogen in thin films, and the embedded porosity in the material is measured using positron annihilation spectroscopy (PAS), a well-established^29–33^ porosimetry technique. The nature and chemistry of the formed structures are characterized by FTIR and Raman spectroscopy.

## Experimental section

### Materials

Spin-on coated low-k thin films have been prepared for this study. The chemicals used for the spin-on organo-silicate glasses were provided by SBA Materials, Inc. The liquid precursor consists of silicon alkoxide esters dissolved in a suitable organic solvent and an amphiphilic block copolymer acting as pore generator^34^. The solute is spin-coated on 6-inch silicon wafers with 2000 rpm for 60 s forming 500 nm thick films at the uncured state before solvent removal. The spin-coated samples are then soft baked for 120 s at 150 °C on a hot plate at ambient air. The soft bake removes the majority of spinning solvent and the tackiness of the film producing films of ~ 486 nm thickness^20^ (thickness shrinkage after FLA as expected from PAS is given in Fig. 2). Then the wafers are cut into small samples of 10 × 10 mm before the curing process.

### Methods

#### FLA

The FLA was performed under continuous flow of N_2_ at normal pressure. The FLA system is composed of twelve Xe-lamps, 30 cm long each, that allows to anneal homogeneously 6 inch wafers using single flash^35^. The maximum temperature obtained during FLA process is limited by the melting point of most materials (e.g. in Si it is about 1400 °C). The short annealing time prevents using conventional pyrometers for contact-free temperature measurements. Therefore, we can only roughly estimate the peak temperature obtained inside the annealed film.

In order to extend our investigations and to be able to put more conclusive statements, both the pulse width and energy density are varied in a controllable way. Here we have used four pulse widths: 1.3, 6, 10, and 20 ms delivering different energy densities as given in Table 1.

**Table 1:** FLA pulse duration and energy density used during FLA curing of spin-on coated low-k.

Pulse width (ms)	1.3	6	10	20
Energy density (J cm^−2^)	30	23, 32, 39	80, 110, 142	30, 50, 70, 95, 125, 142

#### FTIR

Fourier-transform infrared spectroscopy (FTIR) was used to determine the chemical and structural changes after ex-situ annealing at different temperatures. The measurements were performed in transmission mode in the spectral mid-range from 400 to 4000 cm^−1^, using a Bruker Tensor 27 spectrometer. The optical response was given as absorbance after a baseline subtraction. According to the Beer-Lambert law, the absorbance is proportional to the molar concentration of chemical species and the sample thickness. Therefore, all spectra were normalized by the initial thickness in order to quantify changes in bonding arrangements.

#### PAS

Here, we employed two PAS methods; positron annihilation lifetime spectroscopy (PALS) at the Mono-energetic Positron Source (MePS) beamline at HZDR, Germany^36^ and Doppler broadening spectroscopy (DBS) of the annihilation line at the slow positron beamline SPONSOR^37^. In PAS techniques, a positron (e^+^) beam of tunable implantation energy, i.e. implantation depth, is directed to thin films allowing for analyzing atomic defects, voids, and pore contents non-destructively. Implanted e^+^ can form a positronium (Ps) atom in porous structures, a hydrogen-like particle of positron and electron^38^.

In PALS, the lifetime of trapped Ps in pores is shortened from its intrinsic value (142 ns in vacuum) depending on the pore size due to interactions between Ps and electrons on the pore wall (for further details, see sec. 1 in SI). Quantum mechanical models are established to correlate the Ps lifetimes to pores sizes^39–42^. PALS measurements were conducted by using a CeBr_3_ scintillator [51 mm diameter (2″) and 25.4 mm length (1″)] coupled to a Hamamatsu R13089-100 PMT with a µ-metal shield and housed inside a solid Au casing with. An in-house software were used, employing a SPDevices ADQ14-DC-2X-MTCA with 14 bit vertical resolution and 2GS/s horizontal resolution^43^. The overall setup has a timing resolution down to 0.230 ns and a count rate of approximately 10^5^ events/s. The resolution function required for spectrum analysis uses two Gaussian functions with distinct intensities depending on the positron implantation energy, E_p_, and appropriate relative shifts. All spectra contained at least 10^7^ events. All spectra were deconvoluted using the non-linearly least-squared fitting software PALSfit^44^. Conversion of Ps lifetime into pore sizes has been carried out by using EELViS code^45^.

DBS method measures the broadening of the annihilation line of thermalized positron(ium) and electron. DBS is characterized by two parameters S and W, representing the atomic signatures at the annihilation site. The S-parameter is an indicator of the annihilation with valence electrons (low momentum) and it increases with the overall atomic defects including pores. Ps annihilation with high-momentum electrons (core electrons) is represented as the W-parameter, which can qualitatively distinguish between types of atoms around the annihilation spot, e.g. defect or void. In quite large pores, interconnections (escape outside the sample), and in vacuum, Ps annihilates mainly via 3γ while in small pores, 2γ recombination dominates. Therefore, the 3γ/2γ ratio of DBS scales to pore size and interconnectivity. Section S.2 provides more details about DBS and the physics basis behind it. At the source-based SPONSOR system for DBS measurements, positrons have been implanted into a sample with discrete implantation energies E_p_ in the range between 0.05 and 35 keV, which allows for depth profiling from the surface down to couple of micrometers. The parameters S and W defined as a fraction of the annihilation line in the middle (511 ± 0.70 keV) and outer regions (508.56 ± 0.30 keV and 513.44 ± 0.30 keV), respectively. Plotting calculated S as a function of positron implantation energy, S(E), provides depth dependent information. Two-collinear high-purity Ge detectors (energy resolution of 780 ± 20 eV) of the SPONSOR setup have been used to perform coincidence Doppler broadening spectroscopy (cDBS).

#### Raman spectroscopy

The micro-Raman spectroscopy was performed at room temperature using 532 nm laser for the excitation at 10 mW power focused on the spot of 1 µm diameter. The phonon spectra were recorded by liquid-nitrogen cooled Si-CCD in backscattering geometry.

## Results and discussions

### Structural evolution

**Figure 1:**
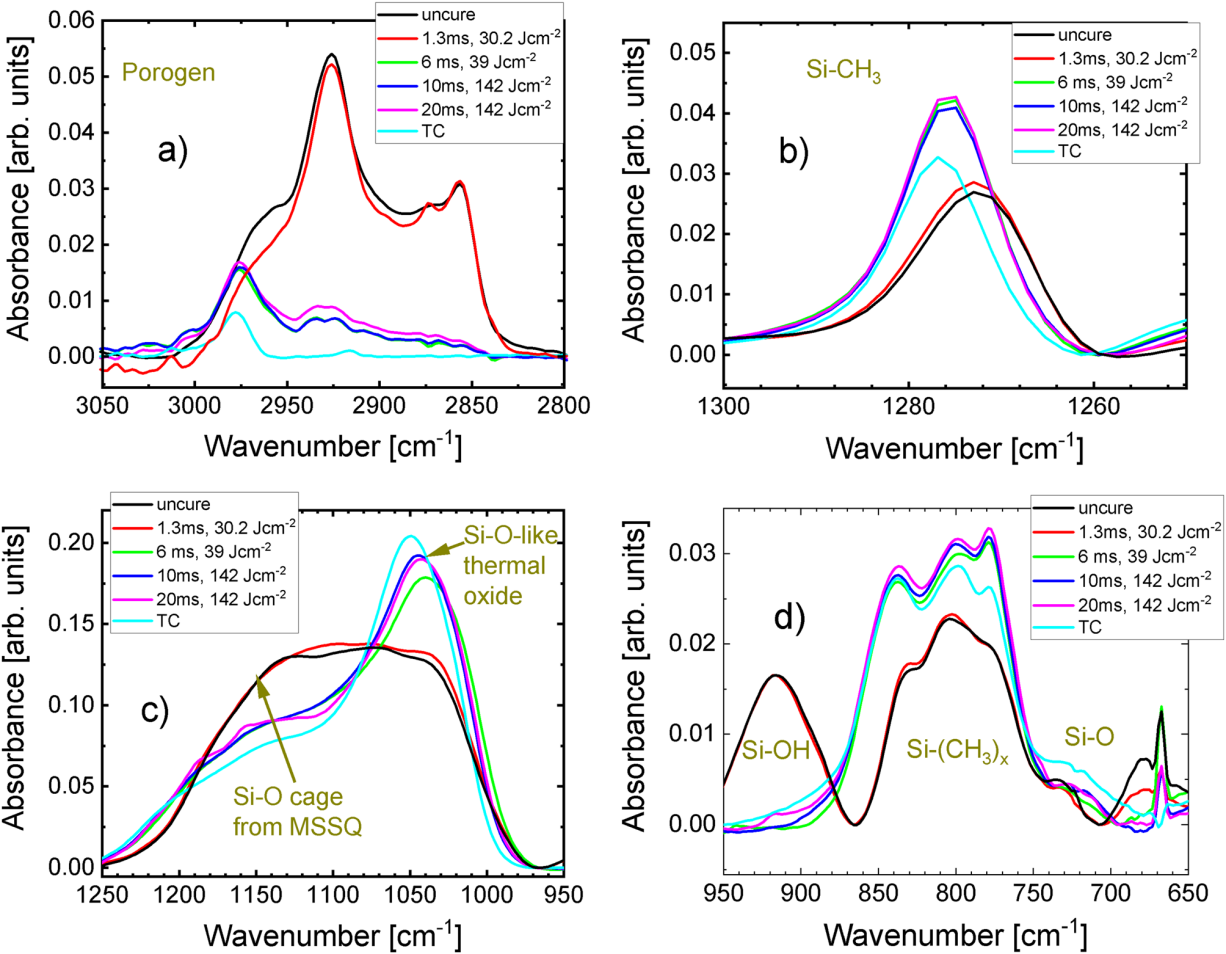
Regions of FTIR spectra of low-k films after FLA at t_*FLA*_ = 1.3, 6, 10, 20 ms with 30.2 J cm^−2^, 39 J cm^−2^, 142 J cm^−2^,142 J cm^−2^ energy densities, respectively of (**a**) porogen, (**b**) Si–CH_3_ groups, (**c**) Si–O, and (**d**) Si–(CH_3_)_x_, Si–OH, and Si–O bonds. The full FTIR spectra and in the region of OH are shown in Fig. S.1.a. Uncured and TC samples are shown for comparison.

As a first step, we investigated uncapped-low-k samples exposed to four FLA pulse widths t_*FLA*_ = 1.3, 6, 10, and 20 ms corresponding to different energy densities (see Table 1) by means of FTIR. The aim was to investigate the impact of FLA on structural evolution of the pores and the matrix. Figure 1 shows typical FTIR spectra at the main identified peak positions for different flash durations and the corresponding highest energy densities (detailed overview is illustrated in Fig. S.1.a). We have foreseen that for all t_*FLA*_ total energy density deposited on the films will be not high enough unless the maximum available flash energy is utilized. Figure S.2.a–d illustrates exemplary results of t_FLA_ = 20 ms at energy densities < 142 J cm^−2^ to highlight the significance of achieving the maximum energy density for decomposing the porogen and constructing a stable matrix as discussed below. In order to evaluate the impact and capability of FLA to produce pores in low-k thin films and to define its optimal settings, thermally cured (TC) film at 450 °C for to 90 min and uncured film were used as references for full curing^20^ and the initial state, respectively. The peak at 3000–2800 cm^−1^^46,47^ (Fig. 1a) indicates the amount of porogen, which should vanishes after efficient curing. The Si–CH_3_ peak lies at ~ 1275 cm^−1^^48^ (Fig. 1b). The SiO bonds vibration in Si–O–Si^47^ groups that correlate with the matrix crosslinking structure^20^ (Fig. 1c) appears in the peak between 1250 and  970 cm^−1^. The fingerprint region (950–700 cm^−1^), a complex structure of different Si–(CH_3_)_x_^47^ and Si–O bonds is shown in Fig. 1d. FTIR spectra were compared to the uncured and TC samples to demonstrate the difference in the structures. Porogen signal in Fig. 1a at t_*FLA*_ = 1.3 ms is identical to the uncured sample meaning that 1.3 ms–30 J cm^−2^ is unable to degenerate the porogen. On the other hand, the amplitude of the porogen peak is very low, close to that of the TC sample, at t_*FLA*_ = 6 ms–39 J cm^−2^ and t_*FLA*_ = 10 ms–142 J cm^−2^ (both are almost identical). The porogen peak exhibits a gradual decrease with energy density at t_*FLA*_ = 20 ms (Fig. S.2.a) as it is identical to the uncured sample at 30 J cm^−2^ then it reaches the lowest height, and close to the TC sample, at 142 J cm^−2^. Thus, one can conclude that the lowest amount of porogen residual is found for the highest reachable energy density for t_*FLA*_ = 6, 10, and 20 ms. Given that the removal of porogen is dependent on both the light penetration range and heat dissipation, which is strongly dependent on porosity, layer thickness, etc., it is likely that the heat absorbed at t_FLA_ = 6 ms with modest power density was high enough to effectively remove the porogen, similar to t_FLA_ = 10 ms and 20 ms. Si–CH_3_ absorption (Fig. 1b), which describes the network arrangement, in the FLA samples at t_*FLA*_ = 6 ms–39 J cm^−2^, t_*FLA*_ = 10 ms–142 J cm^−2^, and t_*FLA*_ = 20 ms–142 J cm^−2^ (and energy density > 70 J cm^−2^ in Fig. S.2.b) is higher than that of the TC sample. Presence of Si–CH_3_ terminal groups keep the structure hydrophobic, which is an essential characteristic to preclude moisture adsorption that will increase the k value^24^. This could be beneficial because grafting methyle groups to the pore wall to maintain the hydrophobicity is already being performed in the semiconductor industry^49^. The hydrophobicity of FLA-treated samples can be seen from the peak of OH-bond absorption. The OH-bonds either belong to the network oligomers (hydrogen bond of silanols) or absorbed water. In Fig. S.1.b, the hydrogen bond of silanols contributes significantly to the peak of OH-bonds in FLA samples for t_FLA_ = 1.3 ms–30 J cm^−2^ and 20 ms at power density < 95 J cm^−2^, which represents a very subpar cross-linking process. The peak of the OH-bonds declines in the hydrogen bond of silanols range but extends to the physical absorbed water range for 6 ms–39 J cm^−2^ and 20 ms–95 and 125 J cm^−2^ meaning that these samples absorb water from the atmosphere. Importantly, the OH-bond peak has vanished for 10 ms–142 J cm^−2^ and 20 ms–142 J cm^−2^ indicating no further water uptake. This can be indeed due to a hydrophobic structure because of excess Si–CH_3_ terminal groups or a consequence of a physical barrier (cap layer or isolated pores) preventing water intrusion. The latter is possible the case in 10 ms–142 J cm^−2^ as discussed below in the PAS results. Although the amount of Si–CH_3_ absorption is similar to that observed at 10 ms–142 J cm^−2^ and 20 ms–142 J cm^−2^, suggesting similar hydrophobicity, the detection of physically absorbed water at 6 ms–39 J cm^−2^ remains ambiguous. However, the Si–CH_3_ terminal methyl groups also disrupt the degree of cross-linkage of Si atoms in the matrix deteriorating the mechanical properties^50^. Such a drawback in mechanical stability is common in spin-on organosilsesquioxanes low-k dielectric thin films^12^. Some approaches have been proposed in order to improve the mechanical stability of spin-on low-k thin films including (1) post-deposition curing to create more bridging bonds between the silicon atoms or (2) the replacement of oxygen atoms between Si atoms by carbon-based bridges^24,12,51^. Since the current work is not focused on the assessment of physical properties like mechanical stability, our future efforts will be dedicated in the direction of (1) and (2). The matrix cross-linking for t_*FLA*_ = 10 ms–142 J cm^−2^ and t_*FLA*_ = 20 ms–142 J cm^−2^ (Fig. 1c) reaches almost the TC conditions, which indicates that the matrix structure is similar to the one after TC. The matrix cross-linking is slightly weaker at t_*FLA*_ = 6 ms–39 J cm^−2^ and t_*FLA*_ = 20 ms–125 J cm^−2^ (Fig. S.2.c) and it is much weaker at t_*FLA*_ = 20 ms at lower energy densities (Fig. S.2.c) until approaching the initial uncured state at 30 J cm^−2^ similar to t_*FLA*_ = 1.3 ms–30 J cm^−2^. The lower amount of Si–O bonds and higher of Si–CH_3_ bonds in the FLA samples (Fig. 1d and Fig. S.2.d) indicate, again, slightly lower matrix connectivity.

### Porosity by PAS

As discussed in the FTIR section, flashing at the highest achievable energy density at t_*FLA*_ = 6 ms, 10 ms, and 20 ms is important in order to get the lowest porogen residual, better matrix cross-linking, and higher hydrophobicity. The lowest concentration of porogen is also visible in Fig. S.3 where the W-parameter (see sec. S.2 for more details about DBS and experimental details) after flashing at the highest energy densities at all used t_*FLA*_ is closer to that of the thermally cured sample, while it is identical to the uncured state at t_*FLA*_ = 1.3 ms and 20 ms–30 J cm^−2^. Therefore, the PAS results of FLA-treated samples with the highest energy densities at the mentioned t_*FLA*_ will be discussed as the most representative. Moreover, the uncured sample (untreated state), t_*FLA*_ = 1.3 ms–30 J cm^−2^ (initial case of FLA), and TC (fully cured state) will be also presented.

**Figure 2:**
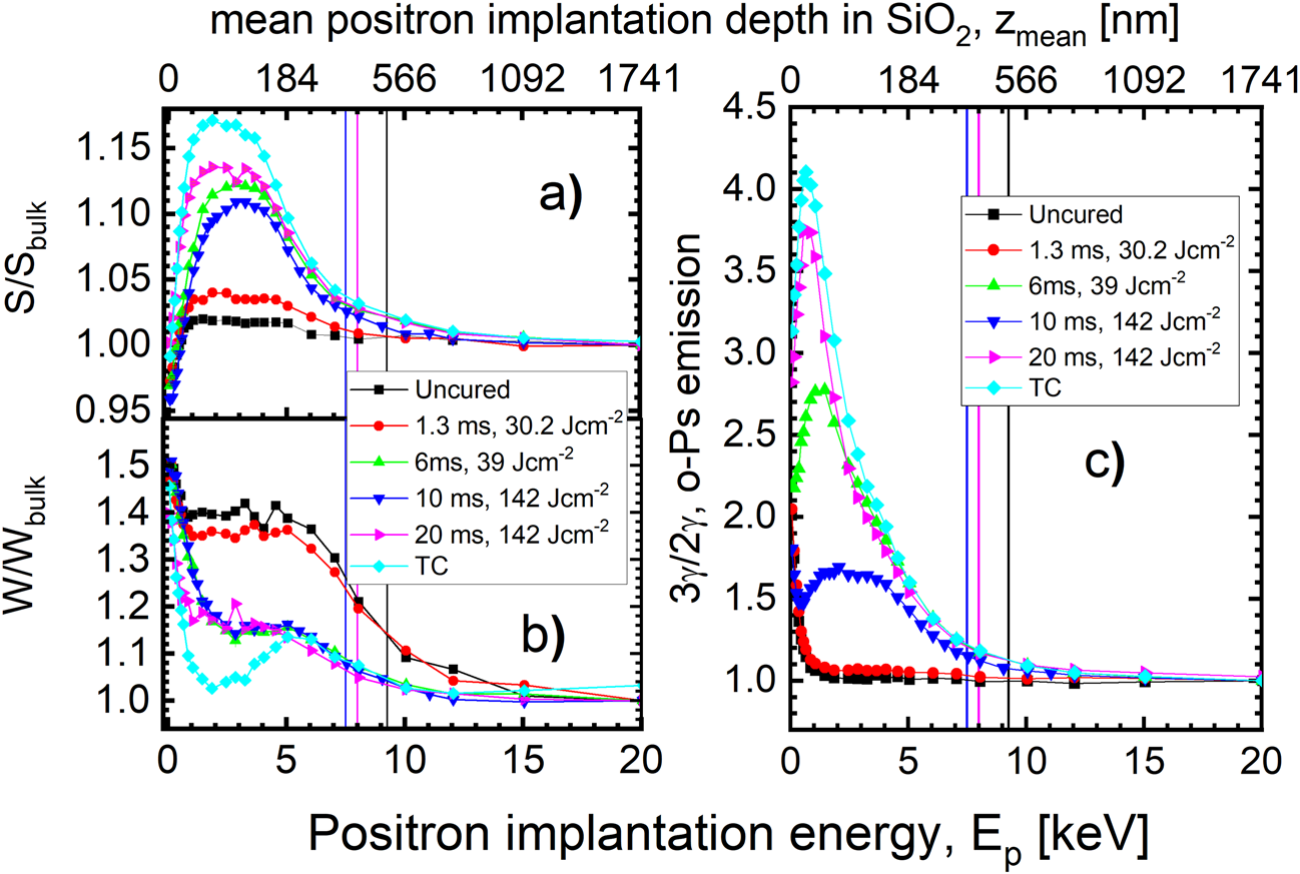
Normalized to bulk (**a**) S- and (**b**) W-parameters and (**c**) o-Ps emission probability 3γ/2γ of low-k thin films cured by FLA with different t_*FLA*_ and energy densities compared to the uncured and thermally cured sample as functions of positron implantation energy, E_p_. All samples are uncapped. The black, magenta, and blue lines represent the thickness of uncured sample and 1.3 ms–30.2 J cm^−2^ (500 nm), 20 ms–142 J cm^−2^ and 6 ms–39 J cm^−2^ (395 nm), and 10 ms–142 J cm^−2^ (355 nm), respectively.

DBS results in Fig. 2a,b show that t_*FLA*_ = 1.3 ms does not provide sufficient energy density to decompose the porogen as the S- and W-parameters are similar to the uncured sample with a slight difference due to the intrinsic free volume of the polymeric porogen. On the other hand, the S-parameter increases when t_*FLA*_ raises from 6 to 20 ms indicating porogen degradation and formation of pores. However, the values are below the TC sample likely due to possible porogen residues in the FLA samples. The shape of 3γ/2γ (Fig. 2c) suggests that both 6 ms and 20 ms flashes created interconnectivity^20^, which is higher in the case of t_*FLA*_ = 20 ms but it is less than that of the TC sample. It also reveals that the resulting structure has detrimental open-to-surface pores. Surprisingly, the 10 ms pulse width shows a typical shape of 3γ/2γ of capped (closed surface pore) samples^24,52^. This important consequence of 10 ms flash towards forming self-capped pores has been confirmed by Raman spectroscopy (Fig. S.4), which showed that this sample specifically has an insulating graphene oxide layer^53^ on top that acts as an annealing-induced cap layer. The graphene oxide in the coating layer absorbs almost all of the incident light, which explains why the signal of 10 ms–142 J cm^−2^ is different from the other samples, whose signals primarily come from the Si substrate. Worth mentioning, graphene oxide layer is considered as an effective dielectric due to its high mechanical and insulting properties^54–56^.

To ensure that the outcomes would be replicated and the cap layer would hold up even with additional thermal treatments, we performed FLA at 10 ms–142 J cm^−2^ on two additional samples that had been preheated at 200 and 250 °C. These two temperatures are higher than the already applied soft back temperature (150 °C). The shape of 3γ/2γ for capped systems is reproduced, according to DBS results in Fig. S.5.a, which means that the cured-induced cap layer is formed despite preheating. The declined values of 3γ/2γ in the preheated samples relative to the as flashed sample can be understood from the porogen signal captured by FTIR in Fig. S.5.b, which demonstrates that the amount of porogen left (integrated area under the peak) in the preheated samples is higher. This leads to smaller formed pores and lower 3γ/2γ values. During preheating, the matrix most likely begins to accumulate and obstruct the pathways for effective porogen removal once FLA is applied.

**Figure 3:**
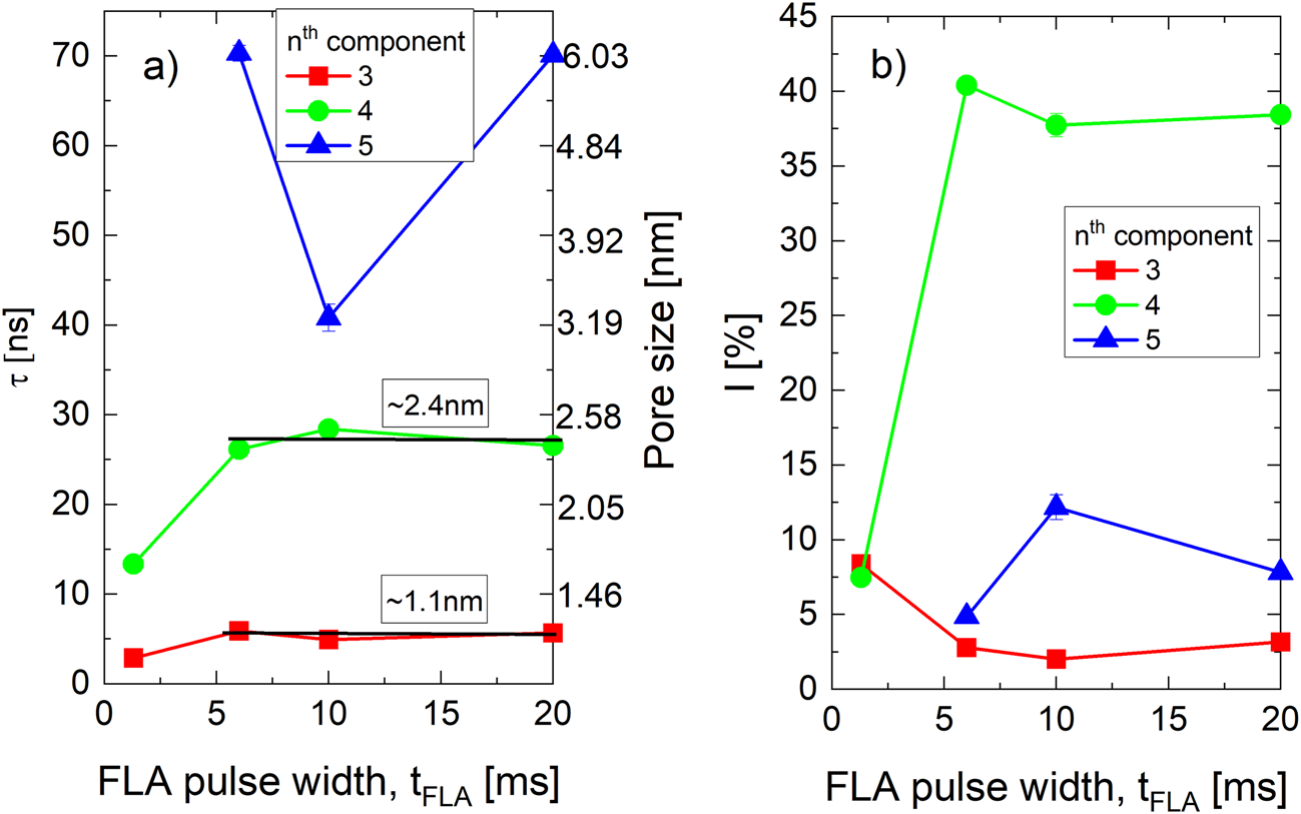
(**a**) o-Ps lifetimes and (**b**) their intensities of matrix, micro- and mesopores, and large mesopores (or interconnections) of uncapped low-k cured by FLA as a function of FLA pulse width t_*FLA*_ at energy densities given in the experimental section and at E_*p*_ = 3.2 keV. Prior to positron measurements, samples have been annealed at 200 °C for 20 min in order to desorb water. The plot includes error bars, but they are of the same size as the data points and may be difficult to see.

In order to determine the pore sizes created after FLA, we performed PALS analysis, which provided five different annihilation states (n = 1–5): τ_n_ and I_n_, where τ represents the lifetime and I is the intensity of the n^th^ component of Ps (or e^+^) in a certain *void or pore (defect)* state. The origins of these five components are distinguished based on the lifetime value and thus revealing the defect or pore size wherein e^+^ or Ps annihilate. Therefore, the components reflect the annihilation of: (n = 1) p-Ps—see sec. S.1- (τ_1_ ~ 0.125 ns); (n = 2) free e^+^ (τ_2_ ~ 0.3–0.5 ns); (n = 3) o-Ps formed inside the matrix and small microspores (τ_3_ ~ 1–5 ns, depending on bond lengths); (n = 4) o-Ps in large micropores and mesopores (τ_4_ ~ 10–30 ns); (n = 5) o-Ps in large mesopores and interconnections (τ_5_ > 40 ns). The intensity values I_n_ reflect the relative occupancy of the e^+^ or Ps in the corresponding annihilation site and it correlates with defect or pore concentrations. Figure 3a depicts the variations of τ_3-5_ (free volume-related components) and Fig. 3b presents their corresponding relative intensities of FLA samples at positron implantation energy E_*p*_ = 3.2 keV. Spherical pore sizes are given on the right axis of Fig. 3a. Besides for t_*FLA*_ = 1.3 ms, both τ_3_ and τ_4_ being nearly independent of t_*FLA*_ measure average matrix free volumes of 1.1 nm and mesopores of 2.4 nm, respectively. The shorter lifetimes of t_*FLA*_ = 1.3 ms indicate smaller pores and likely a larger amount of porogen left as suggested by the FTIR in Fig. 1a and DBS data in Fig. 2. On the other hand, τ_5_ strongly depends on t_*FLA*_. τ_5_ was not detected for t_*FLA*_ = 1.3 ms as likely no mesopores have been created yet. τ_5_ corresponding to ~ 6 nm pore diameter is typical for open and interconnected mesopores was found for t_*FLA*_ = 6 ms and 20 ms while τ_5_ giving mesopores of ~ 3.2 nm was measured for t_*FLA*_ = 10 ms. Therefore, the τ_5_ values indicate that FLA with t_*FLA*_ > 1.3 ms is able to build porosity similar to TC and UV- and plasma- assisted methods. However, the most important finding here is the value of the given pore size (from τ_5_) at t_*FLA*_ = 10 ms. Comparing the 3.2 nm pore size of t_*FLA*_ = 10 ms with the ~ 6 nm at t_*FLA*_ = 6 and 20 ms, suggests that the porogen clustering has decelerated and fewer porogen molecules have been agglomerated to form small and less interconnected pores for t_*FLA*_ = 10 ms. On the other hand, porogen molecules clustered stronger forming bigger and more interconnected pores (see interconnectivity length below) at t_*FLA*_ = 6 and 20 ms. It is worth to mention that the smaller measured pores from τ_5_ at t_*FLA*_ = 10 ms are not associated with the amount of the remaining porogen. In FTIR results (Fig. 1a), one sees that the amount of porogen residual is nearly identical in t_*FLA*_ = 6 ms and 10 ms, but it is higher for t_*FLA*_ = 20 ms. Consequently, if the variation of mesopore size (τ_5_) is related to porogen residuals one expects to get similar pore sizes (lifetimes) for t_*FLA*_ = 6 ms and 10 ms and shorter lifetime in t_*FLA*_ = 20 ms, but this not the case. It seems that the pores for t_*FLA*_ = 10 ms are inherently smaller, irrespective of the porogen content, due to limited porogen clustering which in addition leads to poor interconnectivity. The later assumption is emphasized below where the calculated interconnectivity length is smaller for t_*FLA*_ = 10 ms.

The Ps intensity in the matrix (I_3_) is largest for t_*FLA*_ = 1.3 ms and it decreases and saturates for larger t_*FLA*_. The matrix is still not completely formed at t_*FLA*_ = 1.3 ms thus, the relative intensity I_3_ caries information about free volumes from the fractionally formed matrix and the remaining porogen at the same time (similar lifetimes are expected for both). The contribution from porogen has disappeared at t_*FLA*_ > 1.3 ms. The Ps intensity in the micro-/mesopores (I_4_) increases from ~ 7.5% at t_*FLA*_ = 1.3 ms to 40% at t_*FLA*_ = 6 ms and then it drops slightly to ~ 38% for t_*FLA*_ = 10 ms and 20 ms. These high intensity values are typical for systems with high porosity (Ps intensity scales with pore concentration), which adds another value to FLA as it creates high porosity that is required in low-k. I_5_ (in large mesopores and interconnections) peaks at 12% for t_*FLA*_ = 10 ms and subsequently decreases to 5% and 8% for t_*FLA*_ = 6 ms and 20 ms, respectively. Probably, this is a consequence of the curing-induced cap layer and the semi-isolated pores at t_*FLA*_ = 10 ms pulse width as more Ps are confined within the film (closed porosity) while a significant portion of Ps escapes from the film at t_*FLA*_ = 6 ms and 20 ms pulse widths (open porosity).

To check if the pores at t_*FLA*_ = 10 ms are indeed poorly interconnected, we utilized the method described in^20,49,57^ to calculate the Ps diffusion length or in the other words the interconnectivity length. The method relies on the analysis of 3γ photons as explained below. In systems of interconnected and open-to-surface pores measured under vacuum, the Ps atoms with a thermal velocity of 8 × 10^8^ cm/s^58^ feature lifetimes up to 142 ns. Thus, o-Ps can travel over large distances (up to ~ 8 mm) inside pore networks and they can out-diffuse leaving a thin film of ~ 500 nm thick. These out-diffused Ps atoms annihilate eventually into three photons outside the sample. For a given pore size, the fraction of Ps annihilating by three photons, F_3γ_, will be higher when the interconnectivity length is larger. In order to properly evaluate the F_3γ_ due to Ps out-diffusion, the amount of 3γ annihilation inside the pores are considered and extracted. The fraction of Ps annihilation in mesopores calculated for the same samples is estimated from PALS by fitting the intensity I_5_ (of τ_5_) as a function of positron implantation energy E_*p*_ while the out-diffused fraction of Ps is calculated from the 3γ/2γ ratio of DBS measurements (experimental and fitted F_3γ_ values are depicted in Fig. S.6) as presented in^57^. In Table 2, we present the calculated interconnectivity length, L_*Ps*_, of uncapped low-k cured by FLA at t_*FLA*_ = 6, 10, and 20 ms by assuming film density ρ = 1.9 g cm^−3^. The ratio L_*Ps*_ (FLA) / L_*Ps*_ (TC) reflects the interconnectivity lengths obtained after FLA divided by the 180 nm^20^ interconnectivity length of the same film created by thermal curing at 450 °C for 90 min.

**Table 2:** Interconnectivity length of uncapped low-k thin film cured by FLA at t_*FLA*_ and different energy densities.

Pulse width (ms)	Energy density (J cm^−2^)	Interconnectivity length, L_*Ps*_ (nm)	L_*Ps*_ (FLA)/L_*Ps*_ (TC) (%)
1.3	30.2	0	0
6.0	39.0	69.80	38.7
10.0	142.0	55.64	30.9
20.0	142.0	82.71	45.95

As discussed above, the 1.3 ms flash time provided insufficient energy density to degrade the porogen and pores were not created. The 3γ/2γ ratio is identical for the uncured state hence, there is no interconnectivity at all. However, the pores at t_*FLA*_ > 1.3 ms are interconnected. Interestingly, the calculated L_*Ps*_ has a minimum at t_*FLA*_ = 10 ms with a value of ~ 56 nm in comparison with ~ 70 nm at t_*FLA*_ = 6 ms and ~ 83 nm at t_*FLA*_ = 20 ms. This emphasizes the discussion of the lifetime results and it confirms that the pores created in the sample annealed for t_*FLA*_ = 10 ms are less interconnected with respect to pores made after annealing for t_*FLA*_ = 6 ms and 20 ms. Thus, not only self-sealed surface pores (3γ/2γ in Fig. 1c) are expected at t_*FLA*_ = 10 ms but also the internal pores could be isolated to some extent. The ratio L_*Ps*_ (FLA) / L_*Ps*_ (TC) indicates that none of the FLA films developed a comparable interconnectivity lengths to TC and the maximum ratio is only ~ 46% for 20 ms. This can be attributed probably to the amount of porogen residuals and/or the smaller porogen agglomeration during FLA.

**Figure 4:**
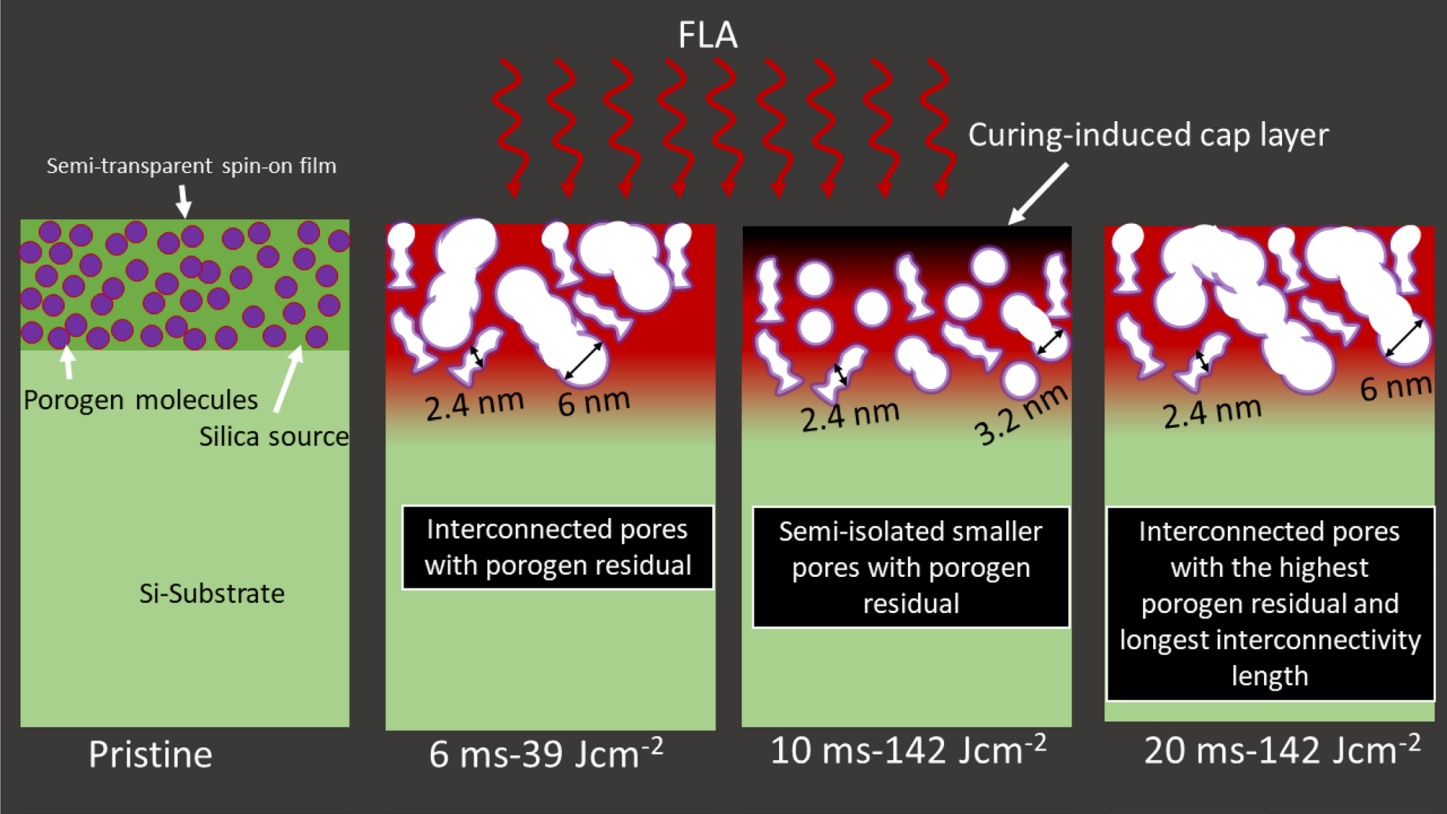
Sketch showing the evolution of the porous structure of low-k thin films as anticipated from positron results after FLA at t_*FLA*_ = 6, 10, and 20 ms at different energy densities.

Combining DBS, PALS, and L_Ps_ results, we can propose the sketch in Fig. 4 to visualize the possible porous structure after FLA, which clarifies that the pores at t_FLA_ = 10 are smaller, semi-isolated, and purer with porogen residues.

**Figure 5:**
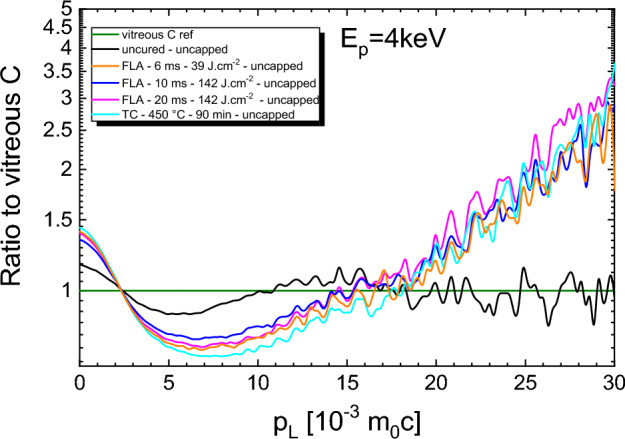
Ratio curves of FLA at t_*FLA*_ = 6, 10, and 20 ms at energy densities of 39, 142, 142 J cm^−2^, respectively measured at E_p_ = 4 keV, obtained by normalization of the other curves by the curve measured from the vitreous carbon reference sample. For comparison, the uncured and fully thermally cured states are shown to represent the initial and final states, respectively.

The final step in this study is dedicated to investigate the chemical fingerprints of the treated films after the FLA process at the annihilation site of Ps. This chemical information can be visualized by coincidence Doppler broadening (cDBS), which evaluates precisely and simultaneously the energy (electron momentum) of the two emitted annihilation photons enhancing the signal-to-noise ratio^59^. The reduced background in cDBS enables better resolution of the relatively rare events of annihilation with core electrons (in the tail of the broadened energy line). Since core electrons are fingerprints of each atom electrical landscape, elemental information are obtained by analyzing the photon intensity in the high-momentum region (similarly to the W-parameter)^20^. In cDBS, a reference sample is used for normalization in order to obtain a ratio between the reference curve and the other samples, which enables to point out similarities or differences. In Fig. 5, the cDBS ratio to the vitreous carbon curve is given for t_*FLA*_ = 6, 10, and 20 ms. The uncured sample represents the initial state and the TC sample serves as the comparison of low porogen residuals and high porosity^20^. In general, the longitudinal electron momentum p_*L*_ < 15 × 10^–3^ m_0_c reflects the combination of free volumes and local chemistry of the polymeric porogen as well as the developed free volume after the curing. At higher momenta, the ratio curves of the reference and uncured sample overlap due to their similar, carbon like electronic structure, at least at the crystal sites where positrons annihilate (open and free volumes). In our previous study^20^ we have shown that the TC sample has almost no porogen and the matrix is well-densified. Hence, the similar shape and magnitude of the ratio curves in case of FLA samples compare to the TC sample suggest slightly larger but still relatively low porogen content as well as already developed matrix. The increase (decrease) of the ratio magnitude around p_*L*_ = 10 × 10^–3^ m_0_c (7–8 × 10^–3^ m_0_c) scales with the porosity of the film at E_*p*_ = 4 keV. In this p_*L*_ region the t_*FLA*_ = 10 ms sample is relative closest to the curve of uncured sample reflecting smaller pore sizes compare to t_*FLA*_ = 6 and 20 ms as demonstrated in Fig. 3a for the large mesopores. However, the higher p_*L*_ > 15 × 10^–3^ m_0_c part overlaps with the TC cured sample proving the same chemical fingerprint.

## Conclusions

In summary, we present a study, which emphasizes on the ability of flash lamp annealing to degenerate pore surfactant in thin films in a highly controlled manner. We employ the method to generate porosity in low-k thin films as an example. Flash lamp annealing therefore may serve as a new and unique technique to be considered as an alternative curing method for thin films. The unquestionable advantage compared to the other curing techniques is a much shorter curing time. Not only that but also our results indicate that FLA at t_*FLA*_ = 10 ms provides well-densified, hydrophobic, self-sealed, and semi-isolated porous structure with low porogen residuals. Consequently, we believe that our results would trigger the attention to FLA as a beneficial curing method and the created porosity would be of great importance, for example, to microelectronics industry. The only challenge of the approach that requires further attention is to employ methods to increase the low matrix linkage of the cured films appearing likely as a consequence of Si–CH_3_ groups. This challenge will be explored in future works aiming at the optimization of FLA parameters (larger pulse width and energy landscape), including the use of post-heating procedures. Additionally, in order to broaden the method's applicability, our future efforts will be concentrated on using FLA to treat other porous systems.

## Supplementary Information

Supplementary Information.

## Data Availability

All data sets used in this study are available with authors and can be shared by the corresponding authors upon reasonable request.
