# Supplementary material for: Millisecond flash lamp curing for porosity generation in thin films
Source: Sci Rep. 2023 May 12;13:7765. doi: 10.1038/s41598-023-34748-x (PMC10181987; doi:10.1038/s41598-023-34748-x)
Supplement: Supplementary file 1 — Supplementary Information. [file 41598_2023_34748_MOESM1_ESM.pdf]

Millisecond Flash Lamp Curing for Porosity Generation in Thin Films

Ahmed G. Attallah [1,2,*], *Slawomir Prucnal* [3], *Maik Buttering* [1], *Eric Hirschman* [1], *Nicole Koehler* [4], *Stefan E. Schulz* [4], *Andreas Wagner* [1], *and Maciej O. Liedke* [1,*]

[1] Helmholtz-Zentrum Dresden-Rossendorf, Institute of Radiation Physics, 01328, Dresden, Germany

[2] Physics Department, Faculty of Science, Minia University, 61519, Minia, Egypt

[3] Helmholtz-Zentrum Dresden-Rossendorf, Institute of Ion Beam Physics and Materials Research, 01328, Dresden, Germany

[4] Chemnitz University of Technology, Center for Microtechnologies, 09107, Chemnitz, Germany

1. Positron annihilation lifetime spectroscopy (PALS)

Implanted positrons into insulators of a certain size of free volumes within its matrix, e.g. dielectrics or emitted from surfaces (inner pore walls), can form the hydrogen-like bond state of a positron and an electron known as positronium (Ps) atom[1–3]. Ps has two possible states; para-Ps and ortho-Ps. *p*-Ps is the state with the spins of the electron and positron are antiparallel and it decays by two photons emission with an intrinsic vacuum lifetime of 0.125 ns. The o-Ps is the state with parallel spins and decays into three photons in vacuum with a characteristic lifetime of 142 ns. Due to its much longer lifetime than *p*-Ps, *o*-Ps can diffuse from bulk to pores owing to its lower zero-point energy in pores. Trapped *o*-Ps interacts with the pore wall until the positron

evolved in *o*-Ps annihilates with an electron from the pore wall with opposing spin or it intrinsically annihilates in three gamma quanta. The former process is known as *pick-off* annihilation and it causes a reduction in o-Ps lifetime depending on the pore size. The dependency of o-Ps lifetime on sizes of small spherical pores was firstly described by the Tao-Eldrup (TE) model and later expanded [4,5] for larger pores including excited states. In materials containing distinct pore sizes, the *o*-Ps annihilation in each pore group contributes an additional o-Ps lifetime component. If the pores are interconnected or open to the surface, the *pick-off* annihilation rate decreases and o-Ps lifetime approaches its vacuum value, 142 ns. These features can be exploited to check for open pores in FLA ULK thin films as if they have any open or interconnected pores, one should see this in o-Ps lifetime. A typical lifetime spectrum N(t) is described by $N(t)=\Sigma\ (1/\tau_i)\ I_i\ \exp(-t/\tau_i)$, where τ_i and I_i are the positron lifetime and intensity of the i-th component, respectively ($\Sigma I_i=1$). All spectra were deconvoluted into five discrete lifetime components. The first two components, τ_1 and τ_2, are related to annihilation of *p*-Ps and unbounded positrons in the matrix. The corresponding relative intensities reflect to a large extend concentration of each defect type (size) as long as the size of compared defects is in the similar range. In general, positron lifetime is directly proportional to defects size, i.e., the larger the open volume, the lower the probability it takes for positrons to annihilate with electrons [6,7]. The positron lifetime and its intensity has been probed as functions of positron implantation energy E_p or in the other words implantation depth (thickness).

2. Fourier-transform infrared spectroscopy (FTIR)

Fig.S.1.a presents the FTIR spectra revealing a detailed overview of the FLA-induced material changes. Thermal curing (TC) and uncured states are shown for comparison.

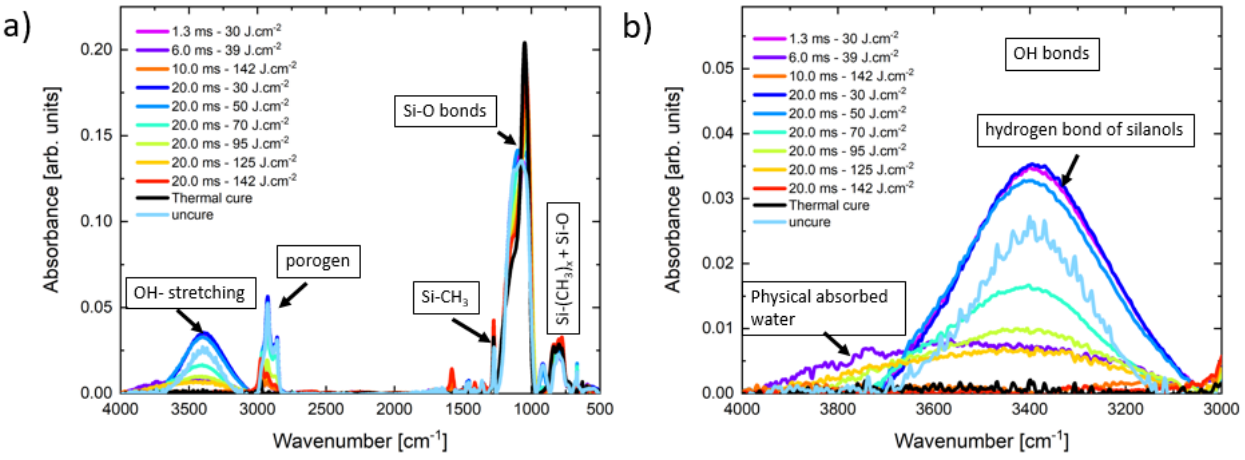

Figure S.1. a) Complete FTIR spectra with peak origins and b) FTIR in the region of water absorption (OH bonds) of FLA, TC, and uncured samples.

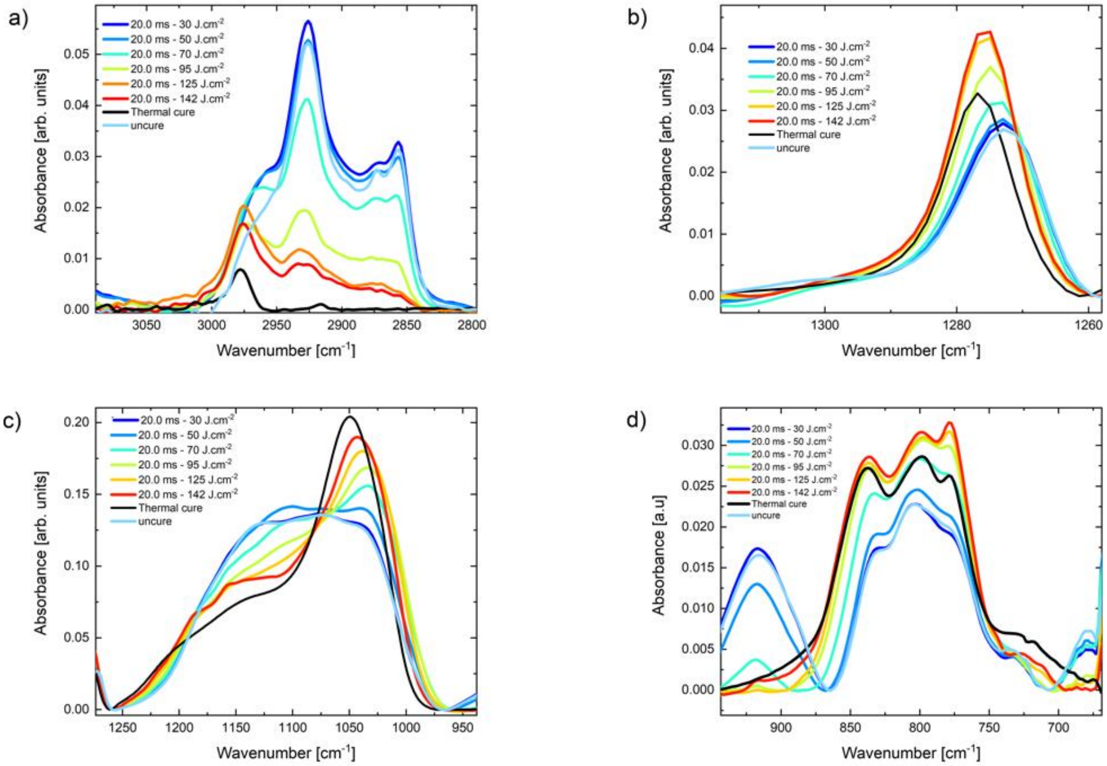

Figure S.2. FTIR signal peaks of a) porogen, b) Si-CH_3 groups, c) Si-O, and d) Si-(CH3)x, Si-OH, and Si-O bonds of low-k films after FLA at tFLA = 20 ms with different energy densities. Uncured and TC samples are shown for comparison.

3. Doppler broadening of annihilation line

Thermalized positrons and Ps are considered to have zero momenta; therefore, any momentum measured during annihilation is associated solely to the electrons momenta at the annihilation site. Electronic momenta result in an energy broadening of the annihilation energy line around 511 keV; the rest mass energy of electron or positron. This energy broadening is measured by Doppler broadening spectroscopy (DBS) which is characterized by two shape parameters, S and W. The S-parameter is a measure of counts of annihilation events in the central region of the photopeak divided by the counts under the broadened peak area. The W-parameter represents the counts in the wings (tails) of the spectrum divided by the total area below the peak [6]. In defective sites and pores, the electronic density is low and the probability of annihilation with valence electrons is higher than with core electrons. Accordingly, the yield is increased in the central region of the spectrum because of the larger fraction of low momentum electrons (valence electrons) causing a higher S-parameter. On the other hand, the W-parameter describes positron annihilation with core electrons (high momentum) and it characterizes the chemical surrounding at the annihilation site. S- and W- parameters will be used in this study to show the change in relative porosity and porogen removal. Escaped o-Ps from open pores into vacuum will annihilate via 3γ emission while pick-off process produces 2γ emission. The 3γ /2 γ ratio can be used to judge if interconnected pores are created (see Ref. [7] for more details). Doppler broadening variable energy positron annihilation spectroscopy (DB-VEPAS) measurements have been conducted at the slow positron beamline SPONSOR [8]. A mean positron implantation depth can be approximated by a simple material density dependent formula: $\langle z \rangle = 36/\rho \cdot E_p 1.62$, where $\rho = 1.9$ g·cm^{-3}. $\langle z \rangle$ approximates the depth and cannot be treated as an absolute measure because it does not account for positron diffusion. The best estimation it gives for materials with high defect concentrations, hence low positron diffusion length.

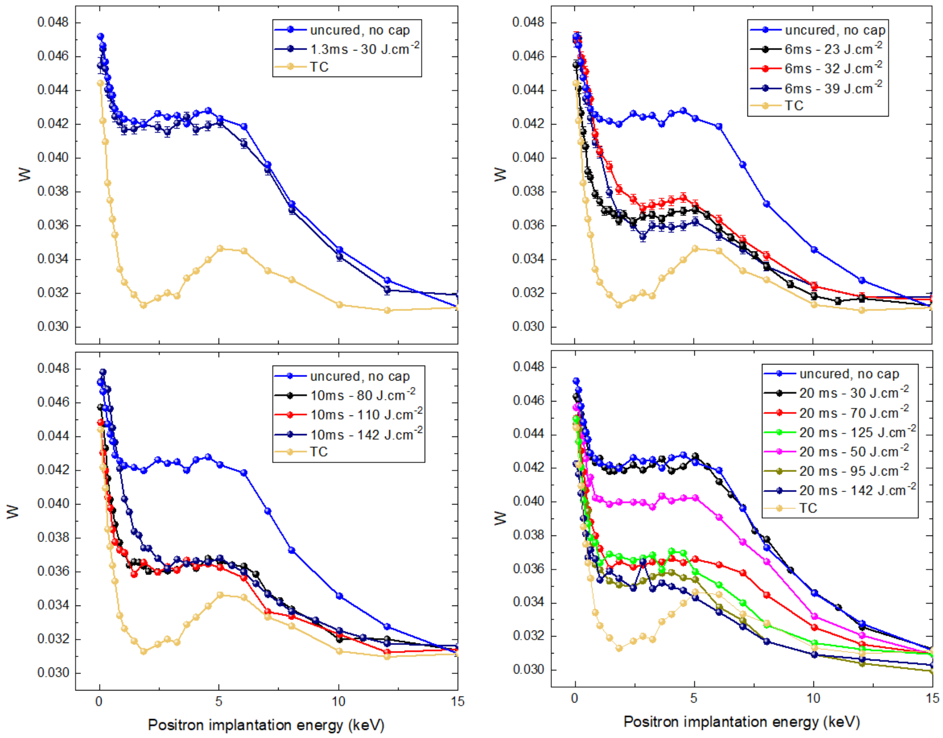

Figure S.3. W-parameter from DB-PAS measurements of FLA at t_{FLA} = 1.3 ms (30 j.cm^{-2}), t_{FLA} =6 ms (23, 32, 39 J.cm^{-2}), t_{FLA} =10 ms (80, 110, 142 J.cm^{-2}), and t_{FLA} =20 ms (30, 50, 70, 95, 125, 142 J.cm^{-2}). Results of uncured sample and TC (450 C - 90 min) sample are shown for comparison

3. Raman Spectroscopy

Raman spectroscopy shows that sample FLA-10ms-142 Jm^{-2} has peaks at wavenumbers > 1200 cm^{-1}, which can be attributed to graphene oxide layer [9]. These peaks are invisible in the other FLA samples and the formed pores are open. The origins of the other peaks in Raman spectra shown in Fig. S.3 are: (i) peak at 303 cm^{-1} is 2TA-Si second transvers acoustic phonon mode in Si, (ii) peak at 520 cm^{-1} is TO/LO in Silicon transvers optical phonon mode, (iii) peaks at about 950 cm^{-1} is the second harmonic of Si peaks.

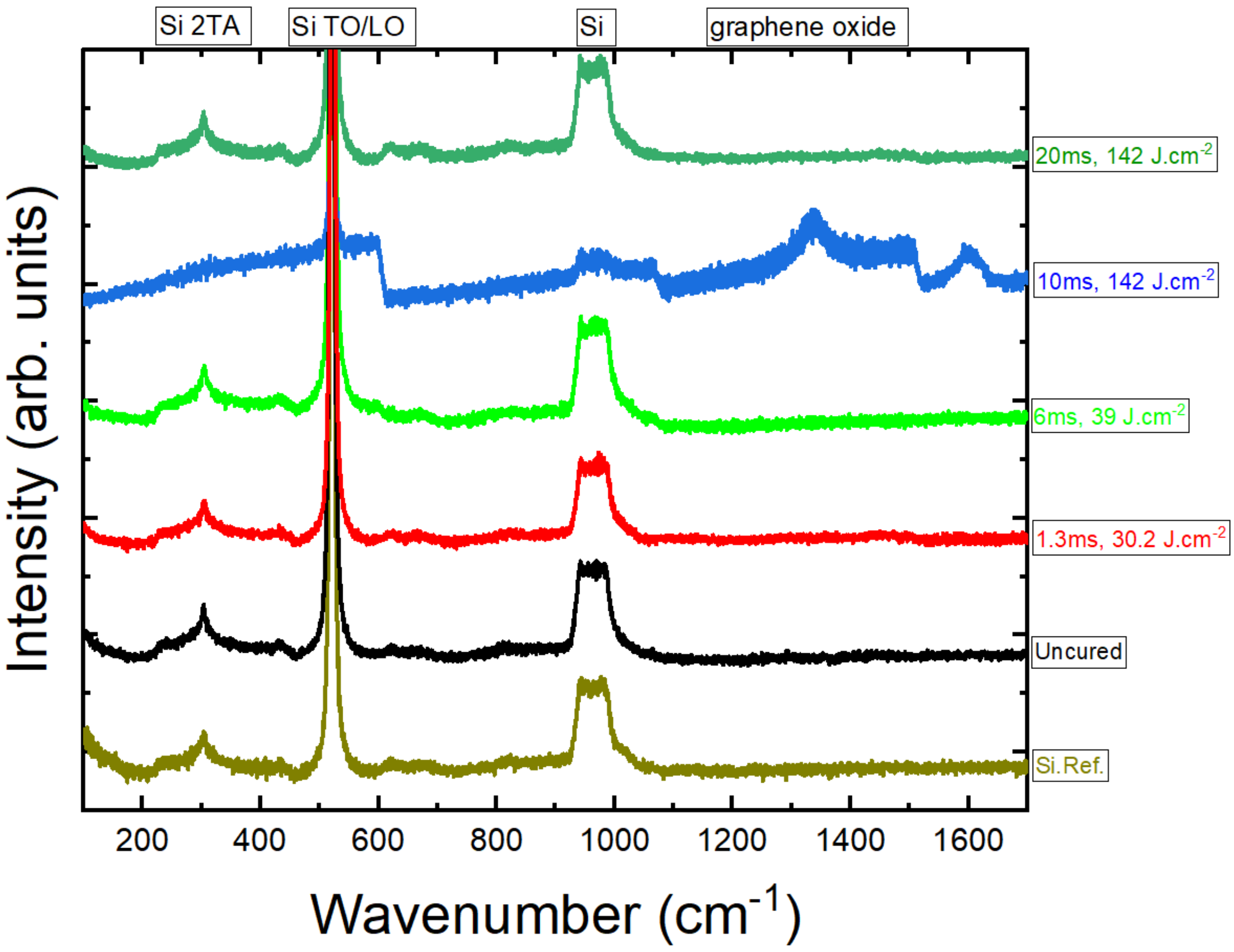

Figure S.4. Raman spectra of FLA samples at t_{FLA} (ms)/ E_{FLA} (J.cm^{-2}) =1.3/30.2, 6/39, 10/142, 20/142 in comparison with uncured and Si-ref. samples.

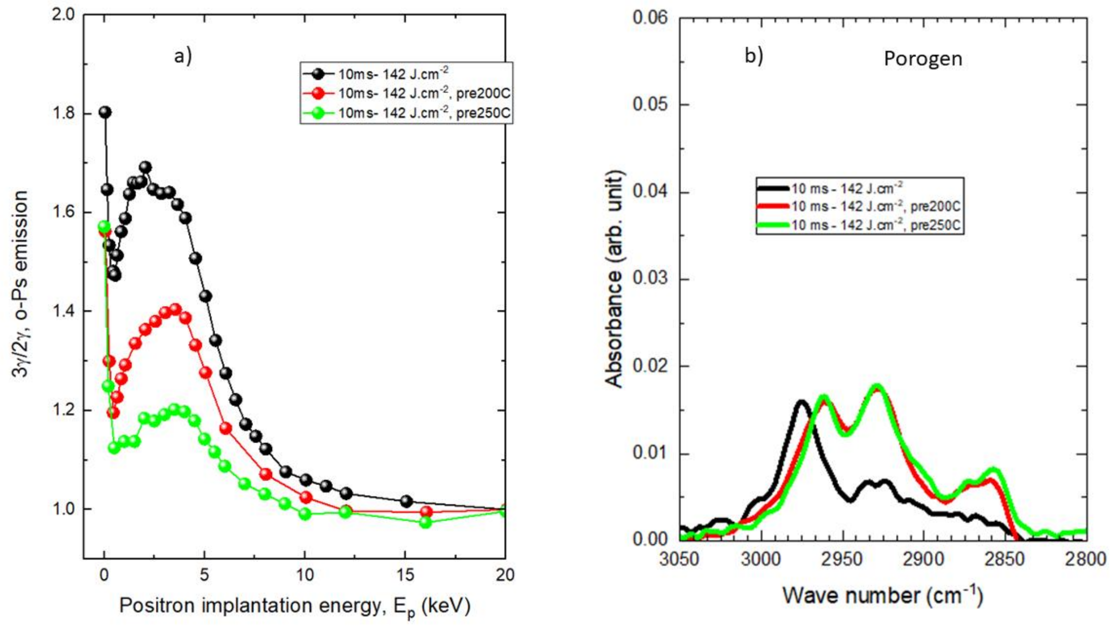

Fig.S.5. (a) Normalized to bulk 3γ/2γ ratio as a function of the positron implantation energy, E_P, and (b) Porogen signal as recorded by FTIR of the as flashed and preheated at 200, 250 ◦C FLA samples at t_{FLA} =20 ms-142 Jm^{-2}

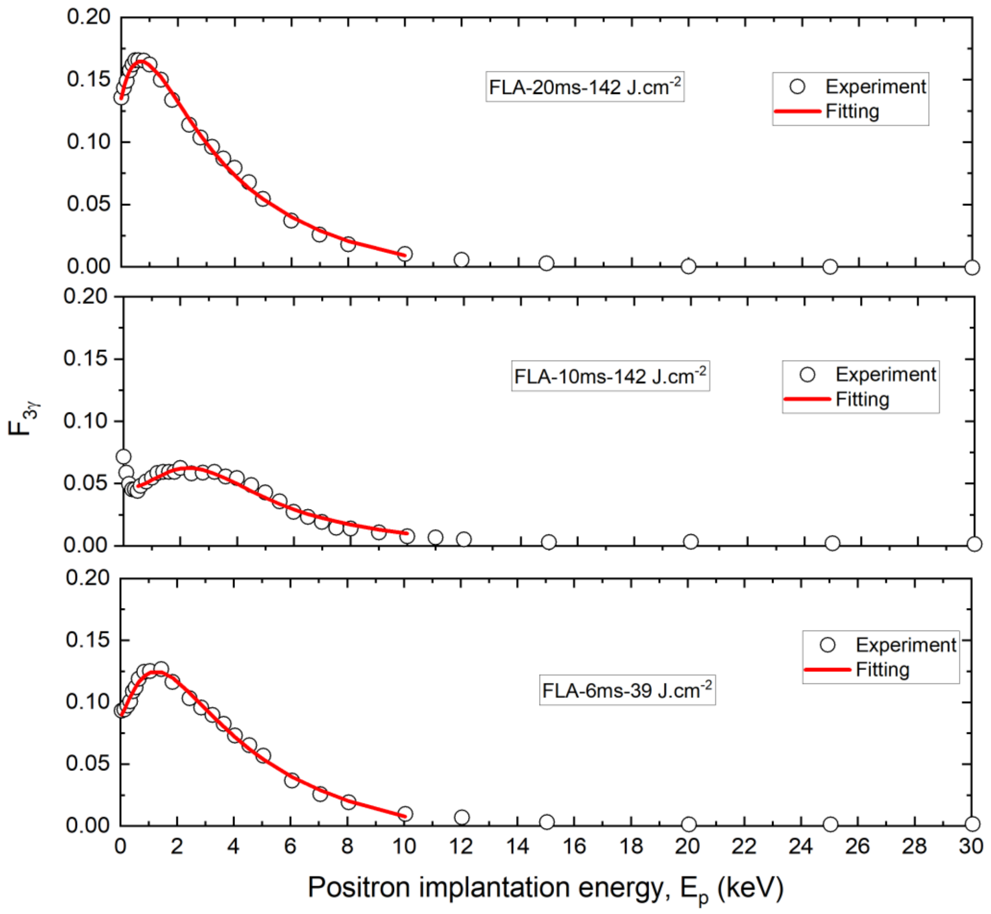

Fig.S.6. Experimental and fitted $F_{3\gamma}$ fraction of uncapped ULK thin films cured by FLA at t_{FLA} =6 ms-39 J.cm^{-2}, t_{FLA} =10 ms-142 J.cm^{-2}, and t_{FLA} =20 ms-142 J.cm^{-2} as a function of the positron implantation energy, E_p.

References

1. Ruark, A. E. Positronium [2]. *Physical Review* vol. 68 278 Preprint at https://doi.org/10.1103/PhysRev.68.278 (1945).

2. Sun, J. N., Gidley, D. W., Hu, Y., Frieze, W. E. & Ryan, E. T. Depth-profiling plasma-induced densification of porous low-k thin films using positronium annihilation lifetime spectroscopy. *Appl Phys Lett* **81**, 1447–1449 (2002).

3. Gidley, D. W. *et al.* Determination of pore-size distribution in low-dielectric thin films. *Appl Phys Lett* **76**, 1282–1284 (2000).

4. Dull, T. L., Frieze, W. E., Gidley, D. W., Sun, J. N. & Yee, A. F. Determination of pore size in mesoporous thin films from the annihilation lifetime of positronium. *Journal of Physical Chemistry B* **105**, 4657–4662 (2001).

5. Zaleski, R., Wawryszczuk, J. & Goworek, T. Pick-off models in the studies of mesoporous silica MCM-41. Comparison of various methods of the PAL spectra analysis. *Radiation Physics and Chemistry* **76**, 243 (2007).

6. Hautojärvi, P. & Vehanen, A. Introduction to Positron Annihilation. in 1–23 (Springer, Berlin, Heidelberg, 1979). doi:10.1007/978-3-642-81316-0_1.

7. A. G. Attallah, N. Koehler, M.O. Liedkea, M. Butterling, E. Hirschmann, R. Ecke, S. & E. Schulz, A. W. Thermal kinetics of free volume in porous spin-on dielectrics: exploring the network- and pore-properties | Request PDF. https://www.sciencedirect.com/science/article/abs/pii/S1387181120304595?dgcid=raven_sd_aip_email doi:https://doi.org/10.1016/j.micromeso.2020.110457.

8. Anwand, W., Brauer, G., Butterling, M., Kissener, H. R. & Wagner, A. Design and Construction of a Slow Positron Beam for Solid and Surface Investigations. *Defect and Diffusion Forum* **331**, 25–40 (2012).

9. Scardaci, V. & Compagnini, G. Raman Spectroscopy Investigation of Graphene Oxide Reduction by Laser Scribing. 48 (2021) doi:10.3390/c7020048.
